# Generation of Recombinant Porcine Parvovirus Virus-Like Particles in *Saccharomyces cerevisiae* and Development of Virus-Specific Monoclonal Antibodies

**DOI:** 10.1155/2014/573531

**Published:** 2014-06-19

**Authors:** Paulius Lukas Tamošiūnas, Rasa Petraitytė-Burneikienė, Rita Lasickienė, Artiomas Akatov, Gabrielis Kundrotas, Vilimas Sereika, Raimundas Lelešius, Aurelija Žvirblienė, Kęstutis Sasnauskas

**Affiliations:** ^1^Institute of Biotechnology, Vilnius University, V. A. Graičiūno 8, 02241 Vilnius, Lithuania; ^2^Institute of Microbiology and Virology, Veterinary Faculty of Veterinary Academy, Lithuanian University of Health Sciences, Tilžės 18, 47181 Kaunas, Lithuania

## Abstract

Porcine parvovirus (PPV) is a widespread infectious virus that causes serious reproductive diseases of swine and death of piglets. The gene coding for the major capsid protein VP2 of PPV was amplified using viral nucleic acid extract from swine serum and inserted into yeast *Saccharomyces cerevisiae* expression plasmid. Recombinant PPV VP2 protein was efficiently expressed in yeast and purified using density gradient centrifugation. Electron microscopy analysis of purified PPV VP2 protein revealed the self-assembly of virus-like particles (VLPs). Nine monoclonal antibodies (MAbs) against the recombinant PPV VP2 protein were generated. The specificity of the newly generated MAbs was proven by immunofluorescence analysis of PPV-infected cells. Indirect IgG ELISA based on the recombinant VLPs for detection of PPV-specific antibodies in swine sera was developed and evaluated. The sensitivity and specificity of the new assay were found to be 93.4% and 97.4%, respectively. In conclusion, yeast *S. cerevisiae* represents a promising expression system for generating recombinant PPV VP2 protein VLPs of diagnostic relevance.

## 1. Introduction

Porcine parvovirus (PPV), first isolated from sows in Germany [1], has been found to occur worldwide [2–4]. PPV is the major causative agent in a syndrome or reproductive failure in swine. This syndrome is characterized by stillbirth, mummified fetuses, early embryonic and fetal death, delayed return to estrus, and infertility (abbreviated as* SMEDI*) [5, 6]. PPV is also shown to be an agent able to increase the effects of porcine circovirus type 2 infection in the clinical course of postweaning multisystemic wasting syndrome [7], which is a significant disease in global swine production [8].

Five different groups of porcine parvoviruses (PPV) have been identified: classic PPV (PPV1), PPV2, PPV3 (known as porcine PARV4, hokovirus, or partetravirus), and PPV4 and porcine bocaviruses, which all have substantial genetic divergence [9–12]. Recently, a new parvovirus provisionally proposed to be named as PPV5 was discovered in the United States [13].

Classic PPV has one serotype subdivided into four clinical genotypes (biotypes) according to their pathogenicity. The NADL-8 strain can cause viremia and crosses the placenta to infect fetuses, leading to fetus death [14]. In contrast, nonpathogenic NADL-2 strain is currently widely used as an attenuated vaccine and causes only limited viremia without crossing the placental barrier in experimental infections [15]. The other two groups are the Kresse and IAF-A83 strains, which are associated with dermatitis and enteric diseases, respectively [16].

PPV is a small, nonenveloped virus, assigned to the genus* Parvovirus*. This group of viruses also infects rodents and carnivores and belongs to the Parvoviridae subfamily within the Parvoviridae family [9]. PPV has a negative, single-stranded DNA of about 5 kb with distinct hairpin termini. The genome contains two major open reading frames (ORFs), each located in the same frame of the complementary strand. ORF1 encodes three nonstructural proteins and the structural proteins VP1, VP2, and VP3 are encoded in ORF2. VP1 and VP2 are translated from differently spliced RNAs, whereas VP3 is formed by proteolytic cleavage of VP2 [17, 18].

VP2 consists of an eight-stranded antiparallel *β*-barrel motif with 4 large loops between *β*-strands. These loops are shown to possess many B-cell epitopes and can tolerate insertions [19, 20] that make PPV VP2 a potential antigen carrier and play a key role in PPV diagnosis and immune prophylaxis [21]. The structure of PPV capsid composed of baculovirus system generated recombinant VP2 is available in 3.5 Å resolution (PDB Accession Number 1K3V) [22].

Immunogenic major capsid protein VP2 of PPV has been synthesized in several expression systems including bacteria [21, 23]. PPV VP2 protein expressed using the baculovirus expression vector system was shown to assemble into virus-like particles (VLPs) similar in size and morphology to the original virions. Such VLPs were shown to induce antibodies in immunized pigs [24] and guinea pigs [25]. VLPs generated in baculovirus system exhibit positive immunoreactivity for PPV and are used in most commercial ELISA tests [26]. Most recently, immunogenic PPV VP2 protein was synthesized in yeast* Pichia pastoris* [27]

The formation of recombinant antigenic human parvovirus capsid protein VLPs in* S. cerevisiae* has been recently demonstrated [28, 29]. Regarding costs, yield, and ease of handling, VLP production in yeast represents an alternative to the recombinant baculovirus expression system, which is so far the dominating source of VP2-derived VLPs of parvoviruses [27, 28].

In the current study, we have generated the PPV VP2 protein as VLPs in* S. cerevisiae* expression system, demonstated their structural and antigenic similarity with viral capsids and developed a new indirect IgG ELISA based on the use of PPV VP2-derived VLPs. Moreover, we have developed a panel of PPV VP2 protein-specific monoclonal antibodies and demonstrated their reactivity with PPV-infected cells.

## 2. Materials and Methods

### 2.1. Serum Samples

One hundred and eighty-three swine serum samples from farms in Lithuania (*n* = 160), Romania (*n* = 14), and Ukraine (*n* = 13) were collected in years 2008–2010 and used in this study. Samples were stored at −70°C prior to testing.

### 2.2. Viral DNA Isolation

Viral nucleic acids (NAs) were extracted from porcine kidney cell culture PK-15 (ATCC CCL-33) infected with porcine parvovirus strain NADL-2. NAs were extracted using commercial QIAamp UltraSens Virus kit (Qiagen GmbH, Hilden, Germany) following the manufacturer's manual and stored at −70°C until use.

### 2.3. Cloning and Characterization of PPV VP2 Gene

The PPV VP2 gene was amplified using High Fidelity Enzyme Mix (Fermentas/Thermo Fisher Scientific, Vilnius, Lithuania) directly from extracted NAs using the following pair of primers (IDT, Munich, Germany):

 PPV-vp2-F 5′-TCTACTAGTACAATGAGTGAAAATGTGGAACAA-3′
 PPV-vp2-R 5′-GAGACTAGTCTAGTATAATTTTCTTGGTATAAGT-3′

The primers used for amplification incorporated* Bcu*I site (underlined) for subcloning into the yeast vector pFX7. The thermal cycle conditions were the following: initial denaturation for 3 min at 95°C, followed by 30 cycles of 94°C for 1 min, 55°C for 1 min, and 72°C for 2 min, and then the final elongation at 72°C for 10 min. The PCR amplification product was digested with* Bcu*I and inserted into* Xba*I-linearized and dephosphorylated yeast expression plasmid pFX7 under control of yeast GAL1-10 promoter [30] and confirmed by PCR and subsequent DNA sequence analysis. The nucleotide sequence of the amplified PPV VP2 was compared with those in GenBank using the Basic Local Alignment Search Tool (BLAST). All DNA manipulations were performed according to standard procedures [31] using enzymes and kits from Fermentas/Thermo Fisher Scientific. Recombinant constructs were screened in* Escherichia coli* DH5*α* F′.

### 2.4. Strains, Media, Yeast Transformation, Cultivation, and Protein Purification

Recombinant construct containing PPV VP2 sequence was screened in* E. coli* DH5*α*F′ cells.* Saccharomyces cerevisiae* haploid strain AH22* MATa* (*leu2 his4 pep4*) was used for the expression of PPV VP2 protein. Selection of yeast transformants resistant to formaldehyde was carried out on the YEPD (1% yeast extract, 2% peptone, 2% dextrose, Difco, Sparks, MD, USA) agar supplemented with 5 mM formaldehyde.* S. cerevisiae* transformants were grown in YEPD medium supplemented with 5 mM formaldehyde or in YEPG induction medium (1% yeast extract, 2% peptone, 2% galactose, Difco). Cultivation of transformed yeast cells, expression and purification of PPV VP2 was performed as previously described [32, 33]. After purification, the total protein concentration was determined by the Bradford assay (Roth, Karlsruhe, Germany) with bovine serum albumin (BSA) used as a standard.

### 2.5. SDS-PAGE and Western Blotting Analysis

The samples were boiled in a reducing sample buffer and separated in gel electrophoresis in SDS-Tris-glycine buffer. Proteins were visualized by staining with Coomassie Brilliant Blue (Sigma-Aldrich Co., St. Louis, MO, USA). For Western blotting, proteins were electrotransferred to Immobilon P membrane (Millipore, Bedford, MA, USA) as described by Sambrook and Russell [31]. The membranes were blocked with 5% milk in phosphate buffered saline (PBS) for 2 h. The blocking solution was removed and the blots were incubated with the MAbs against PPV VP2 protein (undiluted hybridoma supernatants). Secondary antibodies conjugated to horseradish peroxidase (HRP) (Bio-Rad, Hercules, CA, USA) were used for detection of specific antibody binding. The blots were stained with 3,3′,5,5′-tetramethylbenzidine (TMB) ready-to-use chromogenic substrate (Clinical Science Products Inc., Mansfield, MA, USA).

### 2.6. Electron Microscopy

After purification by CsCl ultracentrifugation, suspension of the recombinant PPV VP2 protein was placed on 400-mesh carbon coated copper grids (Agar Scientific, Stansted, UK). The protein samples were stained with 2% aqueous uranyl acetate solution (Reachim, Moscow, Russia) and examined with a Morgagni-268 electron microscope (FEI, Eindhoven, The Netherlands).

### 2.7. Characterization of Serum Samples by Commercial Test

Porcine serum samples were assayed for the presence of anti-PPV antibodies using a commercial INGEZIM PPV compact kit (Ingenasa, Madrid, Spain). This is an enzymatic assay based on the blocking ELISA technique which uses MAb specific for porcine parvovirus VP2 protein, and baculovirus expression systems generated recombinant capsid of VP2. The sera were tested according to the recommendations of the manufacturer. Two blocking percentage (BP) values were used for result interpretation: samples with BP higher than 30% were considered as positive and samples with BP lower than 25% were considered as negative. Samples with BP between both values were considered as doubtful.

### 2.8. Indirect ELISA

Polystyrene microtiter plates (Nerbe plus, Winsen/Luhe, Germany) were coated with 50 ng per well of recombinant PPV VP2 protein, diluted in 100 *μ*L of 0.05 M carbonate-bicarbonate coating buffer (pH 9.6) and incubated overnight at 4°C. Plates were washed three times with PBST (phosphate buffered saline with 0.05% (v/v) Tween 20, (Bio-Rad, Richmond, CA, USA)) and then blocked by the addition of 150 *μ*L of blocking buffer per well (1x Roti-Block, Carl Roth GmbH & Co.) and incubation at room temperature for 1 hour. After blocking, the plates were washed three times with PBST and 100 *μ*L aliquots of serum specimens, diluted 1 : 400 in PBST with 1% BSA, were added to the wells. Antigen concentration and serum dilution level for this assay were determined by titration to reach optimal conditions for sensitivity and specificity (data not shown). After 2 h of incubation at 37°C, the plates were rinsed three times with PBST. HRP-conjugated rabbit anti-pig IgG (Sigma-Aldrich Biosciences, Seattle, USA) diluted 1 : 30 000 in PBST, containing 1% BSA, were added to the wells in 100 *μ*L aliquots and incubated for 1 h at 37°C. The plates were washed as described above. Binding of specific antibodies was visualized by the addition of 100 *μ*L/well of TMB substrate (Clinical Science Products Inc., Mansfield, MA, USA). After 10 min of incubation at the room temperature, the reaction was stopped by adding 100 *μ*L/well of 10% sulphuric acid and the optical density (OD) was measured at 450 nm (reference filter 620 nm).

### 2.9. Production of Monoclonal Antibodies

MAbs to recombinant PPV VP2 were produced essentially as described by Kohler and Milstein [34]. Eight-week-old female BALB/c mice (obtained from a breeding colony at the Center for Innovative Medicine, Vilnius, Lithuania) were immunized at days 0, 28, and 56 by a subcutaneous injection of 50 *μ*g of recombinant PPV VP2 protein. For an initial immunization, the antigen was emulsified in complete Freund's adjuvant (Sigma-Aldrich). Subsequent immunizations were performed without an adjuvant, with the antigen dissolved in PBS. Three days after the final injection, mouse spleen cells were fused with Sp2/0-Ag 14 mouse myeloma cells using polyethylene glycol 1500 (PEG/DMSO solution, Hybri-Max, Sigma-Aldrich). Hybrid cells were selected in growth medium supplemented with hypoxanthine, aminopterin, and thymidine (50×HAT media supplement, Sigma-Aldrich). Samples of supernatant from wells with viable clones were screened by an indirect ELISA (as described above) using goat anti-mouse IgG (Bio-Rad) diluted 1 : 5000 to detect specific antibodies to PPV VP2. Hybridomas secreting specific antibodies to PPV VP2 protein were subcloned twice by a limiting dilution assay. Hybridoma cells were maintained in complete Dulbecco's modified Eagle's medium (DMEM, Biochrom, Berlin, Germany) containing 15% fetal calf serum (Biochrom) and antibiotics. Antibodies in hybridoma culture supernatants were isotyped using the mouse monoclonal antibody isotyping kit (Pierce, Thermo Scientific) in accordance with the manufacturer's protocol.

### 2.10. Immunofluorescence Assay

The reactivity of the MAbs with PPV-infected cells was analyzed by immunofluorescence assay (IFA) using porcine parvovirus FA substrate slides (VMRD, Inc., Pullman, USA) containing fixed swine testicle cells infected and noninfected with porcine parvovirus strain KY-11. The slides were treated according to the manufacturer's protocol, incubated with undiluted hybridoma supernatants and developed with fluorescein isothiocyanate- (FITC-) labelled secondary antibody (BD Biosciences, Franklin Lakes, NJ, USA). The immunostained slides were observed by fluorescent microscope Olympus IX-70 (Olympus, Tokyo, Japan).

## 3. Results and Discussion

### 3.1. Expression and Purification of PPV VP2 VLPs in Yeast

**Figure 1:**
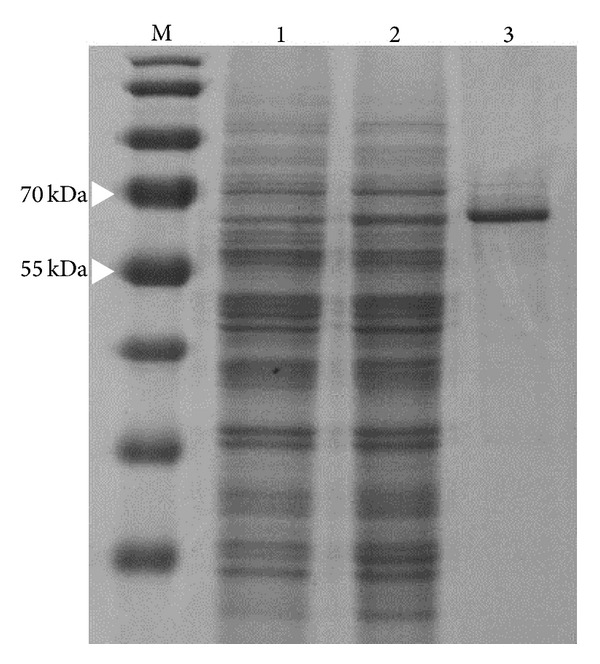
Analysis of* S. cerevisiae* cell lysates and purified PPV VP2 protein by SDS-PAGE. Lysates of* S. cerevisiae* harboring plasmids pFX7 (lane 1) and pFX7-PPV VP2 (lane 2) as well as CsCl-gradient purified PPV VP2 protein (lane 3) were separated in a 12% SDS-PAGE gel and stained with Coomassie Brilliant blue. PageRuler Prestained Protein Ladder (Fermentas/Thermo Fisher Scientific) was used as molecular mass standard in lane M.

The gene encoding PPV VP2 was derived from the nucleic acids extract obtained from PPV-infected cell culture. The 1700 bp sequence amplified by PCR was sequenced and confirmed to be identical to VP2 gene from porcine parvovirus strain NADL-2 (GenBank Entry Number NC001718). The gene was cloned into* S. cerevisiae* expression vector pFX7 under the galactose-inducible promoter. A SDS-PAGE analysis of the lysate of induced yeast biomass revealed a major protein band of approximately 64 kDa (Figure 1, lane 2). No additional protein bands were observed in crude lysates of* S. cerevisiae* harboring empty yeast vector pFX7 (Figure 1, lane 1). After centrifugation of lysates through 30% sucrose cushion, the pellets harboring recombinant proteins were subjected to CsCl-gradient centrifugation. CsCl gradients revealed recombinant PPV VP2 protein (Figure 1, lane 3) in fractions with buoyant density of 1.286–1.308 g/mL.

In several preparative procedures, the yield of purified recombinant PPV VP2 protein was found to be 8-9 mg per liter of induced yeast culture. There was no significant yield difference using fresh or frozen biomass. After CsCl gradient purification, the recombinant VP2 protein was dialyzed against PBS and stored at −20°C in PBS containing 50% glycerol.

**Figure 2:**
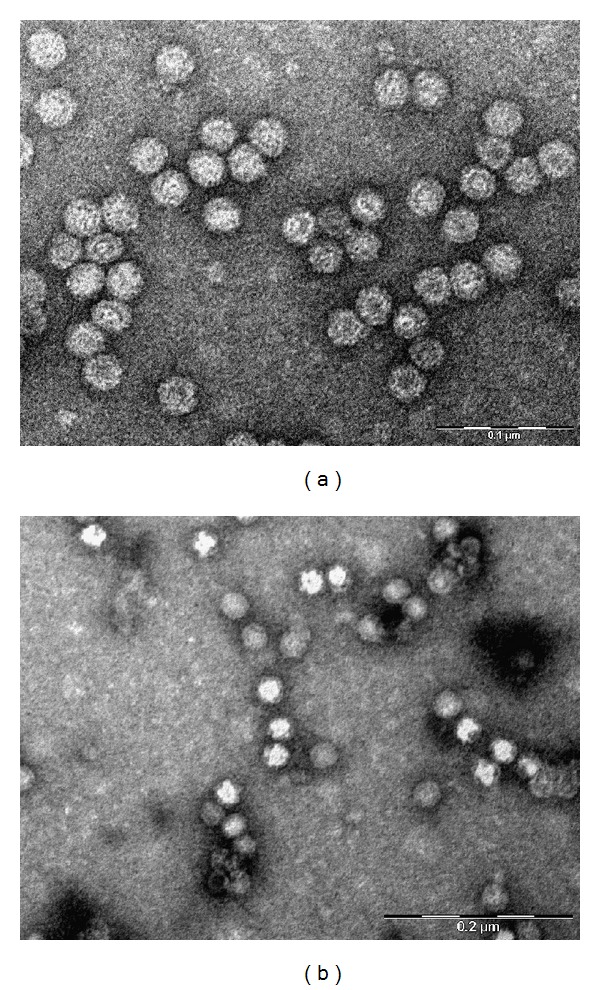
Electron micrograph of recombinant PPV VP2 VLPs in CsCl fraction ((a), scale bar = 100 nm) and VLPs resolubilized after lyophilisation ((b), scale bar = 200 nm).

Formation of VLPs by PPV VP2 protein was confirmed by negative staining electron microscopy. Typical icosahedral structures of parvoviruses with a diameter of approximately 25–30 nm were observed indicating that PPV VP2 protein is self-assembled to VLPs (Figure 2(a)). VLPs of PPV produced in* S. cerevisiae* expression system were similar to those previously generated in insect cells [35] or native PPV particles [17]. Treatment with 25 mM EDTA or 10 mM EGTA did not cause the dissociation of recombinant VLPs indicating that the assembled structures do not require divalent ions (data not shown). The recombinant PPV VP2-derived VLPs were found to be stable when lyophilized and stored at −20°C longer than a year and VLPs remained intact when resolubilized in PBS as no pentamers or disrupted particles were observed by electron microscopy (Figure 2(b)). Moreover, the ELISA results using freshly prepared and resolubilized PPV VP2 antigen were fully concordant (data not shown). The stability of VLPs is crucial to ensure their successful transportation and possible application in the point-of-care tests.

VP2 protein is a major immunogen of most parvoviruses [36, 37]. Therefore, it is successfully employed for the serodiagnostics as well as epidemiological studies of PPV infection [24]. Moreover, VP2 protein is the major agent for developing vaccines [38]. To meet the need for stable recombinant VLPs of PPV, several expression systems were tested as an alternative to baculovirus expression system that is a major source of the antigen for the market [26].* E. coli* [23],* Lactobacillus casei* [21], and recently yeast* Pichia pastoris *[27] were reported to have been successfully used for producing PPV VP2 protein, but VLP formation in these expression systems has not been confirmed. To our knowledge, our study provides the first evidence of stable recombinant PPV VP2 VLPs not produced in baculovirus expression system.

*S. cerevisiae* expression system has been shown to be efficient in producing antigenic VLPs of diagnostic relevance of human parvoviruses [28, 29] and porcine circovirus [39]. Therefore, it represents a potential system to meet the need for VLP-forming antigens to detect and differentiate a number of newly discovered porcine.

### 3.2. Indirect IgG PPV ELISA

PPV VP2 protein-derived VLPs generated in* S. cerevisiae* were used to develop an indirect ELISA for the detection of PPV-specific IgG antibodies in swine serum specimens. In order to test the antigenic properties of yeast-derived VLPs, 187 serum samples were tested using INGEZIM PPV compact test as a gold standard and further retested with the newly developed Indirect IgG PPV ELISA. Both assays were performed in parallel for every serum sample to determine the sensitivity and specificity of the new Indirect IgG PPV ELISA. The cut-off value for the new assay was calculated as the mean OD value of the 39 negative sera (identified with the commercial kit) plus 2 standard deviations ($\overset{-}{x}+2\text{SD}$) resulting in 95% confidence. The mean OD value and SD were 0.150 and 0.090, respectively. Therefore, sera with OD values above 0.330 were considered positive (*n* = 129) and those with OD value below this cut-off were assessed as negative (*n* = 58) in the newly developed Indirect IgG PPV ELISA.

Thirty-eight out of the 39 sera tested as negative with a commercial kit were assessed as negative by the Indirect IgG PPV ELISA. Nine out of 137 positive and all 11 doubtful serum samples by INGEZIM assay showed the OD value below the cut-off in the Indirect IgG PPV ELISA and were considered as negative (Table 1). Thus, the calculated specificity and sensitivity for the new Indirect IgG PPV ELISA were 97.4% (38/39) and 93.4% (128/137), respectively. All 9 false-negative samples of the new assay were weak positive in INGEZIM kit showing blocking percentage in the 33–45% range. All samples above BP equal to 30% were considered positive in this commercial kit. The only false-positive sample in the Indirect IgG PPV ELISA showed OD = 0.354 that is just above the cut-off OD of 0.330. To obtain more precise estimation of the sensitivity and specificity of the new assay, additional evaluation with more serum samples and alternative assays must be done in the future. Alternatively, the precision of the test can be improved using other formats of ELISA. In summary, results of the current study are promising to the use of PPV VP2 antigen synthesized in yeast* S. cerevisiae* in diagnostic kits.

### 3.3. Generation of Monoclonal Antibodies and Their Characterization

Purified recombinant PPV VP2 protein was used to immunize mice and generate PPV VP2-specific MAbs. After screening and cloning of positive hybridoma clones, 9 stable hybridoma cell lines producing IgG antibodies were derived. Six MAbs produced by hybridoma clones were of IgG1 subtype and the remaining three were found to be of IgG2a subtype. All MAbs reacted specifically with recombinant PPV VP2 protein in ELISA and did not react with other yeast-expressed proteins used as a negative control (Table 2).

**Figure 3:**
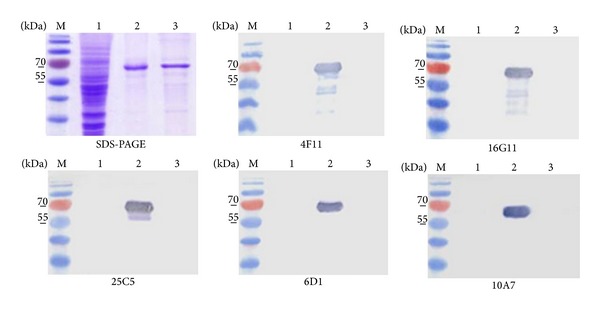
Western Blot analysis of MAbs raised against PPV VP2. Lanes 1: lysates of* S. cerevisiae* harboring plasmids pFX7; lanes 2 and 3: yeast synthesized recombinant PPV and human bocavirus 1 VP2 proteins, respectively. PageRuler Prestained Protein Ladder (Fermentas/Thermo Fisher Scientific) was used as molecular mass standard in lanes M. MAb number used in each WB is indicated below the corresponding blot picture. Only blots for linear-epitope recognizing MAbs are provided.

To characterize the nature of the epitopes recognized by the MAbs, their reactivity in Western blotting was analyzed. The MAbs 4F11, 16G11, 25C5, 6D1, and 10A7 recognized SDS-denatured PPV VP2 protein in Western blotting assay (Figure 3, lanes 2). This result indicates that these MAbs recognize SDS-denatured epitopes of the PPV VP2 protein. The other four MAbs (clones 1F8, 16A1, 22G2, and 23A7) did not recognize SDS-denatured PPV VP2 protein in Western blotting assay (data not shown), suggesting that these MAbs recognize conformation-dependent epitopes.

**Figure 4:**
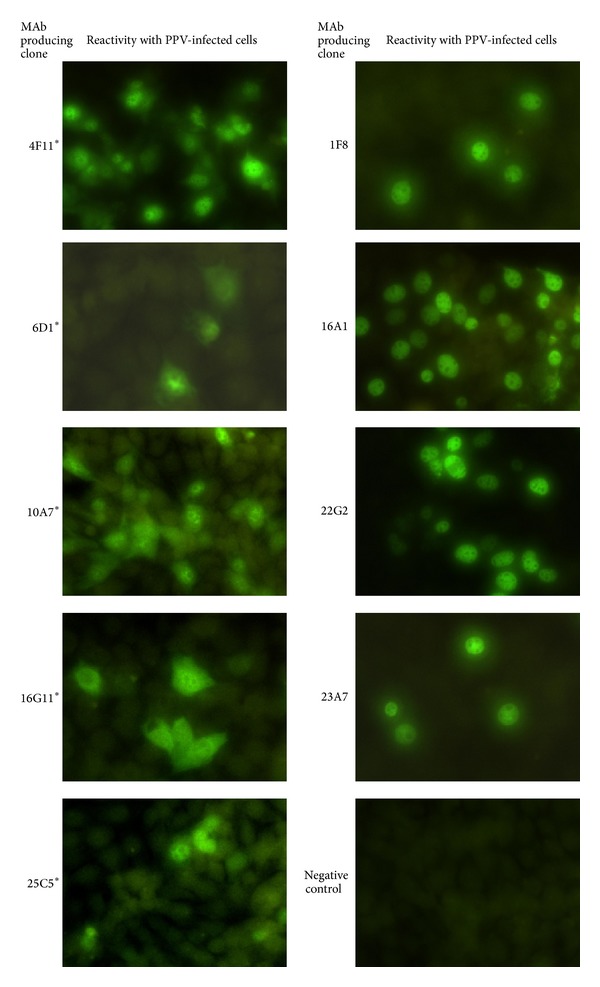
Fluorescence microphotographs showing the reactivity of 9 MAbs raised against yeast-derived PPV VP2 protein with PPV-infected cells on commercial slides (VMRD, Inc.). The codes of the MAbs are indicated on the left side of each picture. The MAbs recognizing linear PPV VP2 epitopes are indicated with an asterisk. As a negative control, negative control serum included in the kit is used.

The specificities of the MAbs were further analyzed by IFA to verify the ability of the MAbs to recognize native virion. For this purpose, commercial porcine parvovirus control slides containing virus-infected and noninfected fixed cells were used. None of the MAbs reacted with noninfected cells, which confirms the specificity of the assay (Figure 4, negative control). Both groups of MAbs recognizing linear or conformational epitopes reacted with infected cells; however, only the latter ones produced images with sharp nucleus-shaped patterns. In contrast, the MAbs recognizing linear epitopes produced signal outside the nuclei but in lesser intensity (Figure 4). This difference could be explained by the possibility that PPV VLPs finish their assembly in the nucleus forming conformational epitopes. Taking into consideration trimer translocation model for other parvoviruses [40], conformational epitopes might be available only in intact capsid but not in trimmers or pentamers formed by VP2 protein. This possibility emphasizes the importance of properly assembled VLPs to elicit strong immune responses when using recombinant antigens as potential vaccines. Further epitope mapping needs to be done to answer if linear epitopes remain accessible on the intact VLP surface or are hidden within the structure. However, our generated MAbs represent an attractive tool for studying intracellular PPV infection and capsid formation process.

The PPV VP2-derived VLPs generated in* S. cerevisiae* have not been yet tested for a possible use as a vaccine in pigs; however, considering results on the antigenic structure and the immunogenicity in mice described in this study, this is an attractive alternative to the currently used recombinant PPV vaccines. In previous studies, PPV VP2-derived VLPs have been shown to be effective epitope carriers to elicit a strong immune response in mice [41, 42]. Furthermore, yeast expression system does not require additional contaminant elimination procedure as described for baculovirus expression system [35] for such recombinant subunit vaccine preparation. Therefore, PPV VP2-derived VLPs generated in yeast* S. cerevisiae* are a promising platform for new PPV vaccine development.

## 4. Conclusions

In this study, we have demonstrated that yeast* S. cerevisiae* is a suitable host for the production of recombinant PPV VP2 protein as stable immunogenic VLPs. The recombinant yeast-derived PPV VP2 protein can be employed in an indirect ELISA for detection of PPV-specific IgG antibodies in swine sera with high specificity and sensitivity. The MAbs raised against yeast-derived PPV VP2 VLPs recognize virus-infected cells and differentiate conformational and linear epitopes of PPV VP2 protein.

## Figures and Tables

**Table 1:** Summary of the concordance of results obtained with the newly developed indirect IgG ELISA and with the commercial INGEZIM test.

ELISA test with recombinant antigen		INGEZIM PPV compact			Total
		Positive	Negative	Doubtful	
Indirect IgG	Positive	128	1	0	129
ELISA test	Negative	9	38	11	58

	Total	137	39	11	187

**Table 2:** MAb isotypes and specificity.

MAb clone	MAb isotype	Indirect ELISA results using yeast *S. cerevisiae *generated antigens		
		Porcine parvovirus VP2	Hantaan (Fojnica) nucleocapsid (N) protein	Tioman nucleocapsid (N) protein
1F8	IgG1	+	−	−
4F11	IgG1	+	−	−
6D1	IgG1	+	−	−
10A7	IgG1	+	−	−
16A1	IgG2a	+	−	−
16G11	IgG1	+	−	−
22G2	IgG2a	+	−	−
23A7	IgG2a	+	−	−
25C5	IgG1	+	−	−

+, OD in ELISA ≥ 1.0; −, no reactivity.
